# Retraction: MicroRNA-424 Is Down-Regulated in Hepatocellular Carcinoma and Suppresses Cell Migration and Invasion through c-Myb

**DOI:** 10.1371/journal.pone.0274145

**Published:** 2022-08-31

**Authors:** The PLOS ONE Editors

Following the publication of this article [1], similarities were noted between this article and articles submitted by other research groups, including [2–6], of which several were previously retracted [7–9].

Similarities included the following figures, which appear to fully or partially overlap, despite being published in different articles and representing different conditions:

- The inhibitor panel in Fig 3B of [1], the inhibitor panel in Fig 2D of [2] and the miR-145 mimics PANC-1 panel in Fig 4C of [3].
- The 0h scramble panel in Fig 3A of [1], and the 0h control panel in Fig 4F of [4].
- The 24h scramble panel in Fig 3A of [1], and the 48h control panel in Fig 4F of [4].
- The pcDNA-c-Myb + miR-424 panel in Fig 5D of [1], and the control-2 panel in Fig 2E of [5].
- The pcDNA + scramble panel in Fig 5D of [1] flipped horizontally and vertically, and the inhibitor panel in Fig 2E of [5].

The corresponding author stated that their article [1] may be similar to others [2–6] because they believe their data may have been used by others without their knowledge or permission as it was not stored securely. They provided data files which do not resolve the concerns regarding similarities between the published articles [1–6].

The unresolved concerns call into question the validity and provenance of the reported results, and the adherence of this article to the PLOS Authorship policy. Therefore, the *PLOS ONE* Editors retract this article [1].

LZ did not agree with the retraction. All other authors either did not respond directly or could not be reached.
